# Complete genome sequence of the newly discovered temperate *Clostridioides difficile* bacteriophage phiCDKH01 of the family *Siphoviridae*

**DOI:** 10.1007/s00705-021-05092-0

**Published:** 2021-05-20

**Authors:** Krzysztof Hinc, Monika Kabała, Adam Iwanicki, Gajane Martirosian, Alessandro Negri, Michał Obuchowski

**Affiliations:** 1 Division of Molecular Bacteriology, Institute of Medical Biotechnology and Experimental Oncology, Intercollegiate Faculty of Biotechnology, University of Gdańsk and Medical University of Gdańsk, Dębinki 1, 80-211 Gdańsk, Poland; 2 Department of Medical Microbiology, Faculty of Medical Sciences in Katowice, Medical University of Silesia, Medyków 18, 40-752 Katowice, Poland

## Abstract

A temperate siphovirus, phiCDKH01, was obtained from a clinical isolate of *Clostridioides difficile*. The phage genome is a 45,089-bp linear double-stranded DNA molecule with an average G+C content of 28.7%. It shows low similarity to known phage genomes, except for phiCD24-1. Genomic and phylogenetic analysis revealed that phiCDKH01 is a newly discovered phage. Sixty-six putative ORFs were predicted in the genome, 37 of which code for proteins with predicted functions. The phiCDKH01 prophage was localized in the host genome. The results of this study increase our knowledge about the genetic diversity of tailed phages.

**Supplementary Information:**

The online version contains supplementary material available at 10.1007/s00705-021-05092-0.

## Introduction

*Clostridioides difficile* is a pathogen with great epidemiological potential that poses a serious threat to human health [1]. In the CDC's latest report on the risk of drug resistance, *C. difficile* was classified as the leading cause of nosocomial infections [2]. *C. difficile* infection (CDI) is closely related to the weakening of the function of the intestinal microbiome as a side effect of antibiotic therapy [3, 4]. CDI is complex and most often manifested with mild, moderate, or severe diarrhea. The development of CDI infection can turn into life-threatening pseudomembranous colitis or toxic megacolon [5–7]. Currently, acute *C. difficile* infection is treated with antibiotics, e.g., metronidazole, vancomycin, or fidaxomicin [8]. The use of antibiotics in the treatment of CDI increases the risk of exacerbation of microflora dysbiosis, causing a reduction or elimination of normal intestinal commensals. Consequently, *C. difficile* may colonize this niche [9]. Moreover, in the case of this infection, antibiotic therapy promotes the recurrence of the disease and increases the chance of emergence of antibiotic resistance [10].

In the last decade, interest in bacteriophages that infect the pathogen *C. difficile* has increased due to their possible contribution to virulence and host biology and their potential as alternative therapeutic agents [11]. So far, all of the phages known to infect *C. difficile* are temperate. In most cases they were isolated from bacterial cells after induction of prophages [12–14]. The described *C. difficile* phages belong to the family *Myoviridae* or *Siphoviridae* of the order *Caudovirales*, i.e., phages with contractile or non-contractile tails, respectively [12, 15]*.* Myoviruses are the most numerous, and their genomes show significant DNA sequence similarity, with a tendency to group into phylogenetically related clusters. In contrast, a limited number of siphoviruses have been described and sequenced, and these phages have been shown to be more genetically diverse [16].

In the current study, a newly discovered phage named phiCDKH01 was isolated and characterized. The phage genome was sequenced and annotated, and phylogenetic analysis indicated that phiCDKH01 is a member of the family *Siphoviridae* and might belong to a novel phage lineage. We also determined the location of the phiCDKH01 prophage in the genome of its host*.*

## Bacterial strain isolation

*Clostridioides difficile* CD34-Sr was isolated from a hospital environment, in the 600-bed clinical hospital of the Medical University of Silesia, Katowice, Poland. The strain was isolated from a bed frame in a patient's room of a nephrology ward. The material was collected using a selective broth enabling the germination of *C. difficile* spores (C diff Banana Broth, Hardy Diagnostics, Santa Maria, USA). After incubation, one loop of broth was replated on the selective *C. difficile* medium chromID *C. difficile* (bioMérieux, Marcy L'Etoile, France) and incubated for 48 hours under anaerobic conditions. Colonies with a characteristic horse odor and yellow-green fluorescence under UV light, microscopically recognized as long, irregular cells with a bulge at their terminal ends, were identified as *C. difficile* using an automated system (VITEK 2 Compact, bioMérieux, Marcy L'Etoile, France).

## Prophage induction and phage isolation

To determine if strain CD34-Sr contained a functional prophage, we used the mitomycin C high-throughput induction method described previously [14]. In this method, the inducible phage DNA in the heated lysate is identified by PCR using specific phage primers targeting the holin genes of myoviruses and siphoviruses [17]. The results confirmed that the amplified PCR product was from the holin gene of an induced siphovirus. We therefore used *C. difficile* CD34-Sr for large-scale phage induction. Mitomycin C induction was performed on 500 ml of log-phase bacteria cultured in BHI broth (Sigma-Aldrich, St. Louis, USA). Following the overnight incubation, the phage lysate was collected, filtered, and concentrated using polyethylene glycol precipitation [18]. We analyzed the concentrated phage lysate under an electron microscope and found only one type of phage particle (Supplementary Fig. S1). The phage fraction was then purified by CsCl gradient centrifugation as described previously [18]. The isolated phage was named phiCDKH01, after its discoverer's initials.

## Genome sequencing and annotation

Genomic DNA of phage phiCDKH01 was purified using a Phage DNA Isolation Kit (Norgen Biotek Corp., Thorold, Canada) following the manufacturer’s instructions. Whole-genome sequencing was performed by Genomed S.A. (Warsaw, Poland) on an Illumina MiSeq platform with 764-fold coverage. High-quality paired-end reads were assembled *de novo* using SPAdes v. 3.13.0 (https://github.com/ablab/spades). The resulting consensus sequence was annotated using myRAST v. 36 (https://rast.nmpdr.org/) [19] and deposited in the GenBank database under accession number MN718463.

## The genomic features of phiCDKH01

**Fig. 1:**
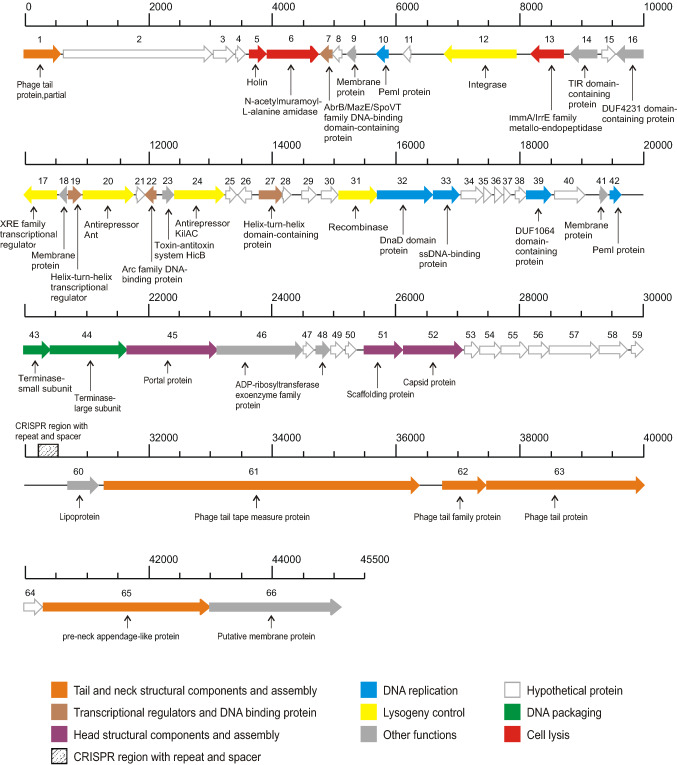
Features of the phage phiCDKH01 genome. The predicted ORFs and their orientations are represented by arrows. The putative functional assignments are indicated below the ORFs. The functional modules were assigned based on gene annotation and genomic organization and are shown in different colours. The position of the CRISPR region with its repeats and spacers is indicated by diagonal hatching.

The genome of phage phiCDKH01 is 45,089 bp in length with a G+C content of 28.7%, which is similar to that of its host *C. difficile*. In the initial annotation, a total of 66 ORFs were identified as probable protein-coding genes. Fifty-three were located on the positive strand, while only 13 were located on the negative strand. No rRNA or tRNA genes were identified. Thirty-seven genes were assigned a predicted function. The complete phage genome could be divided into functional clusters that encode proteins involved in DNA packaging, head and tail morphogenesis, host cell lysis, and replication (Fig. 1). We identified genes for the terminase large subunit (phiCDKH01_44), terminase small subunit (phiCDKH01_43), tail tape measure protein (phiCDKH01_43), two tail family proteins (phiCDKH01_62/63), pre-neck appendage-like protein (phiCDKH01_65), portal protein (phiCDKH01_45), scaffolding protein (phiCDKH01_51), and capsid protein (phiCDKH01_52).

Additionally, we detected genes encoding proteins whose presence confirms the temperate nature of phiCDKH01, including a recombinase (phiCDKH01_31), integrase (phiCDKH01_12), antirepressors (phiCDKH01_20/24), and five putative transcriptional regulators (phiCDKH01_07/17/19/22/27), suggesting that the prophage could affect some bacterial functions.

We identified the gene cluster for host cell lysis containing an *N*-acetylmuramoyl-L-alanine amidase (phiCDKH01_06), a putative holin protein (phiCDKH01_05), and an ImmA/IrrE family metallo-endopeptidase (phiCDKH01_13).

We also found genes involved in DNA replication encoding a DnaD domain protein (phiCDKH01_32), a single-stranded DNA-binding protein (phiCDKH01_33), and two putative PemI proteins (phiCDKH01_10/42). These proteins have been shown to be essential for the autonomous replication of natural plasmids with a low copy number, *e.g.*, R100 [20].

Finally, we identified several additional interesting genes that encode proteins with different functions *e.g.*, an ADP-ribosyltransferase exoenzyme family protein (phiCDKH01_48) that might covalently modify cell actin to alter the physiology of eukaryotic cells in a manner similar to *Clostridium botulinum* C2 or *Clostridium perfringens* E iota toxins [21]. A gene coding for a putative lipoprotein (phiCDKH01_60) might play a role in cortex modification, and thus spore germination [22]. Another gene is predicted to encode HicB antitoxin (phiCDKH01_23), a member of a type II toxin-antitoxin system family found in bacteria and archaea and has been shown to be involved in the stress response, virulence, and persistence [23] (Supplementary Table S1).

Other interesting features of the phiCDKH01 genome include a putative CRISPR (clustered regularly interspaced short palindromic repeats) element and a nearby CRISPR array comprising five spacers of 35, 36 or 37 bp (Fig 1, Supplementary Table S2). Analysis of the CRISPR array revealed that none of the spacers target known *C. difficile* phages. Although spacers 2 (100% identity) and 5 (97.14% identity) have been detected in several other *C. difficile* genomes, spacers 1, 3 and 4 did not match known sequences (Supplementary Table S2). Of note, no other phages were detected in the strain carrying phiCDKH01, suggesting that the CRISPR array in phiCDKH01 might be active and prevent further infection by phages.

## Phylogenetic analysis

**Fig. 2:**
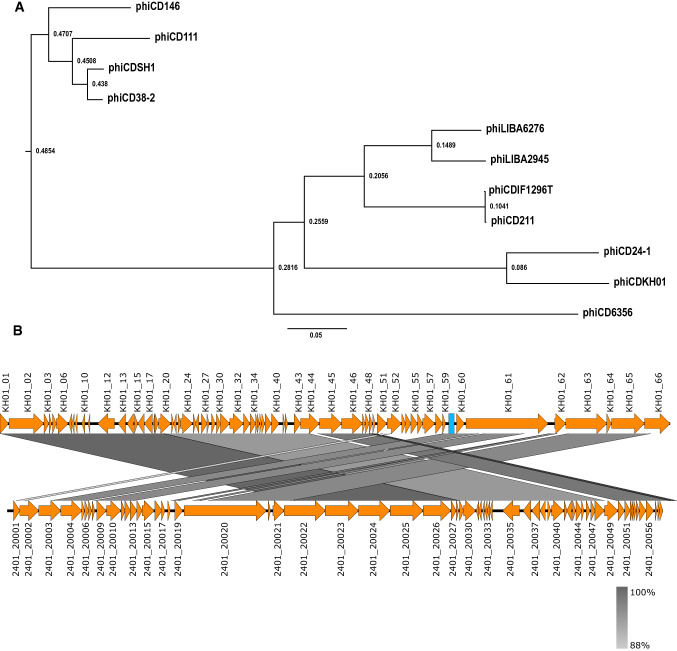
**a** Comparative phylogenetic analysis base on complete genome sequences of *C. difficile* siphoviruses available in the GenBank database. The figure represents the guide tree calculated using the progressive Mauve algorithm. Numbers associated with each branch represent node ages. **b** Comparison of the genome sequence of phage phiCDKH01 (top) with phiCD24-1 (bottom). Predicted ORFs and the direction of transcription are indicated by block arrows. The blue box represents a putative CRISPR element. Conserved regions are shaded in grey. The colour intensity corresponds to sequence identity level (89% to 100%). Genomic comparisons were performed using BLASTn. Similarities with E values lower than 1e-100 are plotted. The figure was produced using Easyfig 2.2.5 [26].

The entire genome sequence of phiCDKH01 was included in a multiple alignment together with genomic sequences of 10 other *Clostridioides difficile* siphoviruses available in the GenBank database. The alignment was performed using Mauve v. 2.3.1 (http://darlinglab.org/mauve/mauve.html) [24], by the progressive Mauve method. The results were visualised using FigTree v. 1.4.4 software (https://github.com/rambaut/figtree) (Fig. 2a). The most closely related phage turned out to be phiCD24-1, which was originally isolated from a clinical isolate exhibiting the 078 PCR ribotype [13, 25]. The sequences of phiCDKH01 and phiCD24-1 share 89% identity and can be considered members of the same genus according to ICTV rules (Fig. 2b).

## Location of the phiCDKH01 prophage in the genome of *C. difficile*

Bacterial genomic DNA of strain CD34-Sr was isolated using an E.Z.N.A. Bacterial DNA Kit (Omega Bio-tek, USA). Whole-genome sequencing was performed using an Illumina MiSeq platform (Genomed S.A.) with 72-fold coverage. After a quality check, the reads were assembled *de novo* in SPAdes v. 3.13.0 to create 70 contigs. These sequences are available in GenBank under accession number JACSDL000000000 and were subjected to automatic annotation. The sequence of phiCDKH01 was found in the contig JACSDL010000003.1 from nt 288,650 to 333,698. The prophage is integrated between the loci H7706_07450 and H7706_07755. H7706_07450 shares sequence similarity with a manganese catalase family protein (GenBank accession no. MBC6710325.1). H7706_07755 is annotated as the *ilvB* gene, coding for the biosynthetic-type acetolactate synthase large subunit (GenBank accession no.MBC6710385.1).

## Supplementary Information

Below is the link to the electronic supplementary material.

Supplementary file1 (PDF 850 KB)
