# Supplementary material for: Complete genome sequence of the newly discovered temperate Clostridioides difficile bacteriophage phiCDKH01 of the family Siphoviridae
Source: Arch Virol. 2021 May 20;166(8):2305–10. doi: 10.1007/s00705-021-05092-0 (PMC8270841; doi:10.1007/s00705-021-05092-0)
Supplement: Supplementary file 1 — Supplementary file1 (PDF 850 KB) [file 705_2021_5092_MOESM1_ESM.pdf]

Complete genome sequence of the newly discovered temperate *Clostridioides difficile* bacteriophage phiCDKH01 of the family *Siphoviridae*

Archives of Virology

Krzysztof Hinc[1*], Monika Kabała[2], Adam Iwanicki[1], Gajane Martirosian[2], Alessandro Negri[1] Michał Obuchowski[1]
[1]Department of Molecular Bacteriology, Intercollegiate Faculty of Biotechnology of University of Gdańsk and Medical University of Gdańsk, Dębinki 1, Gdańsk 80-211, Poland
[2]Department of Medical Microbiology, Medical University of Silesia, Faculty of Medical Sciences in Katowice, Medyków 18, Katowice 40-752, Poland

* corresponding author: krzysztof.hinc@biotech.ug.edu.pl

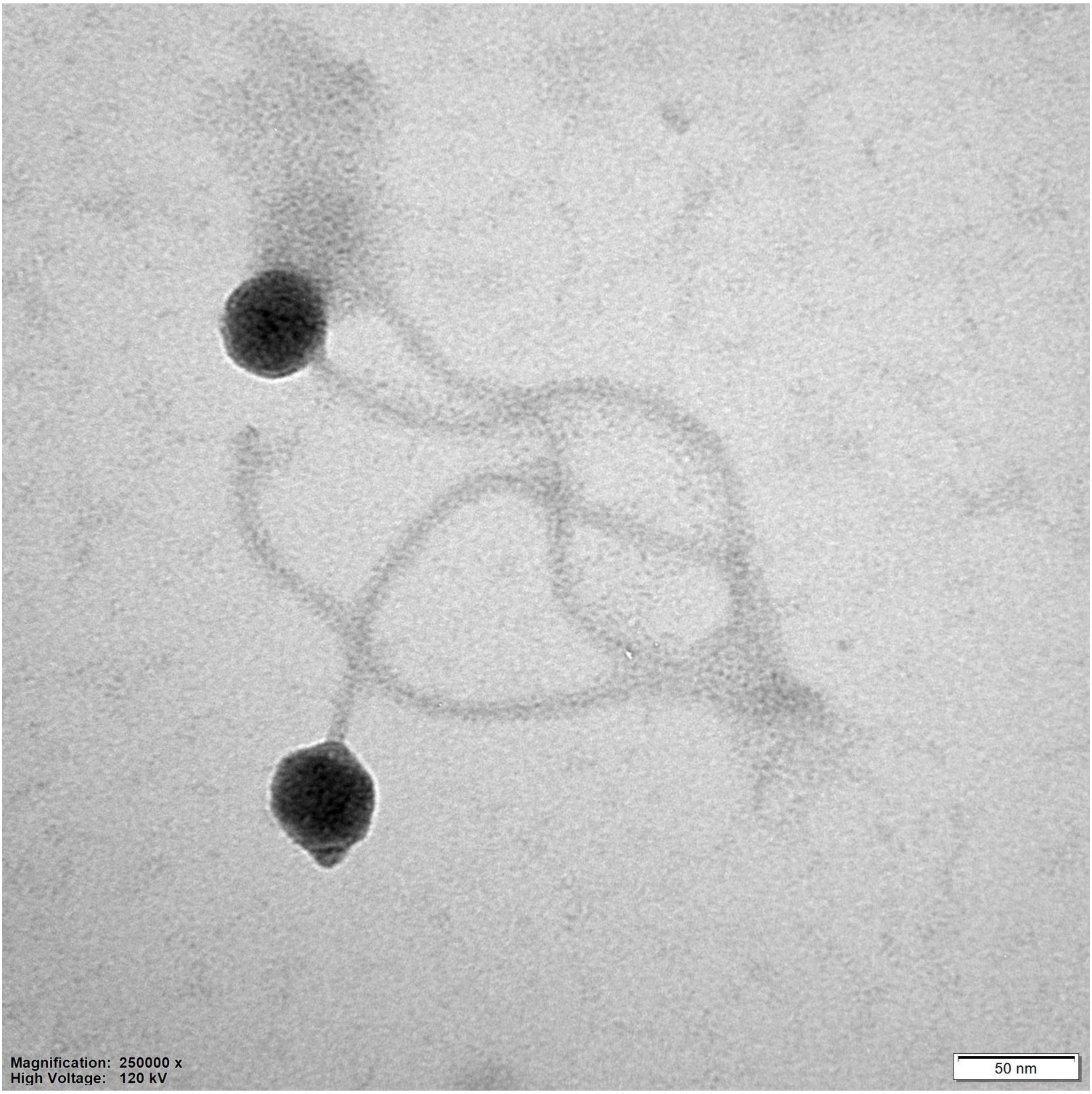

Fig. S1 phiCDKH01 morphology observed using a transmission electron microscope Tecnai Spirit BioTWIN microscope (FEI) at an acceleration voltage of 120 kV. Phage particles were stained with 2% uranyl acetate.

Table S1. ORFs locations, transcriptional orientations, sizes of encoded proteins, possible function, BLAST hit, alignment and E value

Locus tag	Coding region (bp)	Strand	Protein size （aa）	Predicted functional	BLAST hit [source organism][a]	Accession number	Alignment (%ID)	E value
**phiCDKH01_01**	**4-576**	**+**	**190**	**tail protein - partial**	**phage tail protein [*Clostridioides difficile*]**	**MBH6979172.1**	**190/636 (100%)**	**3e-125**
phiCDKH01_02	611-2944	+	777	hypothetical protein	hypothetical protein [*Clostridioides difficile*]	WP_021411116.1	777/777 (100%)	0.0
phiCDKH01_03	2959-3285	+	108	hypothetical protein	hypothetical protein [*Clostridioides difficile*]	WP_021411117.1	108/108 (100%)	2e-69
phiCDKH01_04	3311-3463	+	50	hypothetical protein	hypothetical protein [*Clostridioides difficile*]	WP_021372475.1	50/50 (100%)	3e-25
**phiCDKH01_05**	**3527-3793**	**+**	**88**	**holin**	**putative holin [*Clostridium phage* phiCD24-1]**	**CEK40241.1**	**88/88 (100%)**	**2e-63**
**phiCDKH01_06**	**3728-3504**	**+**	**268**	**N-acetylmuramoyl-L-alanine amidase**	**N-acetylmuramoyl-L-alanine amidase [*Clostridioides difficile*]**	**WP_021411118.1**	**268/268 (100%)**	**0.0**
**phiCDKH01_07**	**4644-4820**	**-**	**58**	**AbrB/MazE/SpoVT family DNA-binding domain protein**	**AbrB/MazE/SpoVT family DNA-binding domain-containing protein [*Clostridioides difficile*]**	**WP_021411119.1**	**58/58 (100%)**	**2e-33**
phiCDKH01_08	4821-4988	-	55	hypothetical protein	hypothetical protein [*Clostridioides difficile*]	WP_021411120.1	55/55 (100%)	2e-28
**phiCDKH01_09**	**5065-5193**	**-**	**42**	**membrane protein**	**putative membrane protein [*Clostridioides difficile* P24]**	**EQJ50015.1**	**42/42 (69.05%)**	**1e-09**
**phiCDKH01_10**	**5517-5702**	**-**	**61**	**PemI protein**	**PemI [*Clostridioides difficile]***	**WP_077725908**	**61/61**	**1e-34**

							**(96.72%)**	
phiCDKH01_11	5931-6059	-	42	hypothetical protein	hypothetical protein QUA_1656 [*Clostridioides difficile* P49]	EQJ91741.1	42/42 (100%)	3e-18
**phiCDKH01_12**	**6577-7707**	**-**	**376**	**integrase**	**site-specific integrase [*Clostridioides difficile*]**	**WP_021411091.1**	**376/376 (100%)**	**0.0**
**phiCDKH01_13**	**7948-8454**	**-**	**168**	**metallo-endopeptidase**	**MULTISPECIES: ImmA/IrrE family metallo-endopeptidase [*Clostridiales*]**	**WP_003429804.1**	**168/168 (100%)**	**1e-114**
**phiCDKH01_14**	**8558-8980**	**-**	**140**	**TIR domain-containing protein**	**TIR domain-containing protein [*Clostridioides difficile*]**	**WP_021365371.1**	**140/140 (100%)**	**2e-95**
phiCDKH01_15	9044-9265	+	73	hypothetical protein	hypothetical protein [*Clostridioides difficile*]	WP_021365372.1	73/73 (100%)	5e-45
**phiCDKH01_16**	**9279-9695**	**-**	**138**	**DUF4231 domain-containing protein**	**DUF4231 domain-containing protein [*Clostridioides difficile*]**	**WP_021365373.1**	**138/138 (100%)**	**9e-90**
**phiCDKH01_17**	**9704-10249**	**-**	**181**	**transcriptional regulator**	**MULTISPECIES: XRE family transcriptional regulator [*Clostridiales*]**	**WP_003433494.1**	**181/181 (100%)**	**6e-128**
**phiCDKH01_18**	**10294-10410**	**-**	**38**	**membrane protein**	**putative membrane protein [*Clostridioides difficile* CD22]**	**EQE30741.1**	**38/38 (100%)**	**3e-14**

**phiCDKH01_19**	**10430-10645**	**+**	**71**	**transcriptional regulator**	**helix-turn-helix transcriptional regulator [*Clostridioides difficile*]**	**WP_021424794.1**	**71/71 (100%)**	**9e-43**
**phiCDKH01_20**	**10676-11494**	**+**	**272**	**antirepressor protein**	**phage antirepressor KilAC domain-containing protein [*Clostridioides difficile*]**	**WP_022583452.1**	**272/272 (100%)**	**0.0**
phiCDKH01_21	11543-11680	+	45	hypothetical protein	hypothetical protein [*Clostridioides difficile*]	WP_165479131.1	45/45 (100%)	1e-22
**phiCDKH01_22**	**11692-11859**	**-**	**55**	**DNA-binding protein**	**MULTISPECIES: Arc family DNA-binding protein [*Clostridiales*]**	**WP_021418310.1**	**55/55 (100%)**	**5e-30**
**phiCDKH01_23**	**11982-12158**	**+**	**58**	**HicB antitoxin**	**toxin-antitoxin system HicB family antitoxin [*Clostridioides difficile*]**	**WP_021424796.1**	**58/58 (100%)**	**1e-33**
**phiCDKH01_24**	**12151-12954**	**+**	**267**	**antirepressor protein**	**phage antirepressor KilAC domain-containing protein [*Clostridioides difficile*]**	**WP_103899391.1**	**267/267 (100%)**	**0.0**
phiCDK01_25	13002-13184	+	60	hypothetical protein	hypothetical protein [*Clostridioides difficile*]	WP_021365376.1	60/60 (100%)	5e-32
phiCDKH01_26	13142-13378	-	78	hypothetical protein	hypothetical protein [*Clostridioides difficile*]	WP_021424801.1	78/78 (100%)	6e-46
**phiCDKH01_27**	**13548-13940**	**+**	**130**	**helix-turn-helix domain-containing protein**	**helix-turn-helix domain-containing protein [*Clostridioides difficile*]**	**WP_021424802.1**	**130/130 (100%)**	**8e-86**

phiCDKH01_28	13927-14070	+	47	hypothetical protein	hypothetical protein [*Clostridioides difficile*]	WP_021390082.1	47/47 (100%)	1e-20
phiCDKH01_29	14237-14479	+	80	hypothetical protein	hypothetical protein [*Clostridioides difficile*]	WP_021365380.1	80/80 (100%)	5e-50
phiCDKH01_30	14558-14839	+	93	hypothetical protein	hypothetical protein [*Clostridioides difficile*]	WP_021365381.	93/93 (100%)	2e-60
**phiCDKH01_31**	**14826-15464**	**+**	**212**	**recombinase**	**recombinase [*Clostridioides difficile*]**	**PCD11566.1**	**212/213 (100%)**	**4e-151**
**phiCDKH01_32**	**15474-16373**	**+**	**299**	**dnaD domain protein**	**dnaD domain protein [*Clostridioides difficile P49*]**	**EQJ91696.1**	**299/299 (100%)**	**0.0**
**phiCDKH01_33**	**16393-16812**	**+**	**139**	**single-stranded DNA-binding protein**	**single-stranded DNA-binding protein [*Clostridioides difficile*]**	**WP_021411097.1**	**139/139 (100%)**	**3e-96**
phiCDKH01_34	16893-16812	+	120	hypothetical protein	hypothetical protein [*Clostridioides difficile*]	WP_021424804.1	120/120 (100%)	9e-82
phiCDKH01_35	16842-17204	+	44	hypothetical protein	hypothetical protein CDIF29637_02647 [*Clostridioides difficile*]	AXU54373.1	44/44 (100%)	2e-21

phiCDKH01_36	17322-17441	+	39	hypothetical protein	hypothetical protein [*Clostridioides difficile*]	WP_021362348.1	39/39 (100%)	6e-19
phiCDKH01_37	17485-17598	+	37	hypothetical protein	hypothetical protein QU3_0844 [*Clostridioides difficile* P42]	EQJ70600.1	37/37 (97.30%)	2e-15
phiCDKH01_38	17617-17814	+	65	hypothetical protein	hypothetical protein [*Clostridioides difficile*]	WP_021408087	65/65 (100%)	4e-39
**phiCDKH01_39**	**17841-18257**	**+**	**138**	**DUF1064 domain-containing protein**	***DUF1064 domain-containing protein [Clostridioides difficile]***	**WP_021362351.1**	**138/138 (100%)**	**2e-89**
phiCDKH01_40	18276-18776	+	166	hypothetical protein	*hypothetical protein [Clostridioides difficile]*	WP_021362352.1	166/166 (100%)	1e-113
**phiCDKH01_41**	**19040-19171**	**+**	**43**	**membrane protein**	**putative membrane protein [*Clostridioides difficile* CD22]**	**EQE30759.1**	**43/37 (100%)**	**6e-15**
**phiCDKH01_42**	**19186-19362**	**+**	**58**	**PemI protein**	**putative PemI [*Clostridioides difficile*]**	**VIB43238.1**	**58/58 (98.28%)**	**9e-32**
**phiCDKH01_43**	**19788-20228**	**+**	**146**	**terminase small subunit**	**terminase small subunit [*Clostridioides difficile*]**	**WP_021365389.1**	**146/146 (100%)**	**1e-99**
**phiCDKH01_44**	**20221-21483**	**+**	**420**	**terminase large subunit**	**PBSX family phage terminase large subunit [*Clostridioides difficile*]**	**WP_021372000.1**	**420/420 (100%)**	**0.0**

**phiCDKH01_45**	**21494-22978**	**+**	**494**	**portal protein**	**phage portal protein [*Clostridioides difficile*]**	**WP_021411100.1**	**494/494 (100%)**	**0.0**
**phiCDKH01_46**	**22978-24396**	**+**	**472**	**ADP-ribosyltransferase exoenzyme family protein**	**ADP-ribosyltransferase exoenzyme family protein [*Clostridioides difficile*]**	**WP_021411101.1**	**472/472 (100%)**	**0.0**
phiCDKH01_47	24389-24565	+	58	hypothetical protein	hypothetical protein [*Clostridioides difficile*]	WP_021365393.1	58/58 (100%)	2e-33
**phiCDKH01_48**	**24606-24836**	**+**	**76**	**ADP-ribosyltransferase exoenzyme family protein**	**ADP-ribosyltransferase exoenzyme family protein [*Clostridioides difficile* P24]**	**EQJ49987.1**	**76/76 (100%)**	**1e-46**
phiCDKH01_49	24837-25052	+	71	hypothetical protein	hypothetical protein [*Clostridioides difficile*]	WP_021365395.1	71/71 (100%)	3e-43
phiCDKH01_50	25082-25258	+	58	hypothetical protein	hypothetical protein [*Clostridioides difficile*]	WP_021365396.1	58/58 (100%)	6e-34
**phiCDKH01_51**	**25394-26029**	**+**	**211**	**scaffolding protein**	**phage scaffolding protein [*Clostridioides difficile*]**	**WP_021372476.1**	**211/211 (100%)**	**8e-149**
**phiCDKH01_52**	**26047-27015**	**+**	**322**	**capsid protein**	**coat protein [*Clostridioides difficile*]**	**WP_131043024.1**	**322/322 (99.69%)**	**0.0**
phiCDKH01_53	27041-27295	+	84	hypothetical protein	hypothetical protein [*Clostridioides difficile*]	WP_021365399.1	84/84 (100%)	2e-54
phiCDKH01_54	27292-27654	+	120	hypothetical protein	hypothetical protein [*Clostridioides difficile*]	WP_021411102.1	120/120 (100%)	1e-80

phiCDKH01_55	27656-28090	+	144	hypothetical protein	hypothetical protein [*Clostridioides difficile*]	WP_021411103.1	144/144 (100%)	1e-102
phiCDKH01_56	28098-28442	+	114	hypothetical protein	hypothetical protein [*Clostridioides difficile*]	WP_021365402.1	114/114 (100%)	2e-76
phiCDKH01_57	28448-29254	+	268	hypothetical protein	hypothetical protein [*Clostridioides difficile*]	WP_021372459.1	268/268 (100%)	0.0
phiCDKH01_58	29313-29789	+	158	hypothetical protein	hypothetical protein [*Clostridioides difficile*]	WP_021411104.1	158/158 (100%)	3e-108
phiCDKH01_59	29782-29979	+	65	hypothetical protein	conserved protein of unknown function [*Clostridium phage* phiCD24-1]	CEK40228.1	65/77 (100%)	1e-37
**phiCDKH01_60**	**30718-31251**	**+**	**177**	**lipoprotein**	**lipoprotein [*Clostridioides difficile*]**	**WP_021411109.1**	**177/177 (100%)**	**7e-127**
**phiCDKH01_61**	**31357-36885**	**+**	**1842**	**tail tape-measure protein**	**phage tail tape measure protein [*Clostridioides difficile*]**	**WP_021411110.1**	**1842/1842 (100%)**	**0.0**
**phiCDKH01_62**	**37323-38078**	**+**	**251**	**tail family protein**	**phage tail family protein [*Clostridioides difficile*]**	**WP_021411111.1**	**251/251 (100%)**	**0.0**

**phiCDKH01_63**	**38080-40839**	**+**	**919**	**tail protein**	**phage tail protein [Clostridioides difficile]**	**WP_021411112.1**	**919/919 (100%)**	**0.0**
phiCDKH01_64	40826-41131	+	101	hypothetical protein	hypothetical protein [Clostridioides difficile]	WP_021411113.1	101/101 (100%)	7e-69
**phiCDKH01_65**	**41149-43368**	**+**	**739**	**pre-neck appendage-like protein**	**phage pre-neck appendage-like protein [Clostridioides difficile]**	**VIF60740.1**	**739/739 (96.48%)**	**0.0**
**phiCDKH01_66**	**44414-45076**	**+**	**220**	**membrane protein**	**putative membrane protein [Clostridioides difficile P53]**	ERM48055.1	**220/169 (96.45%)**	**1e-92**

[a]Only the most relevant hit is shown

Table S2. CRISPR spacer analysis

A. phiCDKH01 CRISPR-spacer sequences

spacer1:	cattattaagtcaaatgttgatgtaactgaacttaa
spacer2:	agtatatctaatataaatgagttcttagttaaccc
spacer3:	tggacgagtatatctgaaggtgttagtagtgcttg
spacer4:	gaattgaatgataaacaaaaaaaagcatgtaatgaac
spacer5:	gctcataataaccataagcgcttttggttaaatttaa

B. Spacer match on published C. difficile phage genomes

phiCDKH01	5 Hits	Length	Start	End	E value
**spacer1**	100%	36	30230	30265	1e-08
**spacer2**	100%	35	30296	30330	3e-08
**spacer3**	100%	35	30361	30395	3e-08
**spacer4**	100%	37	30426	30462	3e-09
**spacer5**	100%	37	30493	30529	3e-09
phi027	No Hit				
phiC2	No Hit				
phiCD111	No Hit				
phiCD119	No Hit				
phiCD146	No Hit				
phiCD24-1	No Hit				
phiCD27	No Hit				
phiCD38-2	No Hit				
phiCD481-1	No Hit				
phiCD505	No Hit				
phiCD506	No Hit				
phiCD52	No Hit				
phiCD6356	No Hit				
phiCDHM1	No Hit				
phiCDHM11	No Hit				
phiCDHM19	No Hit				
phiE16	No Hit				
phiMMP01	No Hit				
phiMMP02	No Hit				
phiMMP03	No Hit				
phiMMP04	No Hit				
phiSemix9P1	No Hit				
phiCD630-01	No Hit				
phiCD630-02	No Hit				
phiLIBA2945	No Hit				
phiLIBA6276	No Hit				

C. Spacer match on published C. difficile strain genomes*

**spacer2**	1 Hit
CP028529.1	100%
CP019858.1	100%
CP016104.1	100%
CP019469.1	100%
LK933005.1	100%
LK932396.1	100%
LK932486.1	100%
CP028361.1	100%
FN665652.1	100%

CP029154.1	100%
CP020424.2	100%
**spacer5**	1 Hit
CP028529.1	97.14%
CP019858.1	97.14%
CP019469.1	97.14%
LK933005.1	97.14%
LK932396.1	97.14%
LK932486.1	97.14%
CP049958.1	97.14%
CP028361.1	97.14%
FN665652.1	97.14%

*removing phiCDKH01 containing strains
